# Assessment of New Molecular Entities Approved for Cancer Treatment in 2020

**DOI:** 10.1001/jamanetworkopen.2021.12558

**Published:** 2021-05-28

**Authors:** Claire E. P. Smith, Vinay Prasad

**Affiliations:** 1 Hematology and Medical Oncology, Boston University School of Medicine, Boston, Massachusetts; 2 Department of Medicine, University of California, San Francisco; 3 Department of Epidemiology and Biostatistics, University of California, San Francisco

## Abstract

This cross-sectional study of all hematology/oncology drugs approved by the US Food and Drug Administration in 2020 assesses the functioning of the drug approval process following disruptions related to the COVID-19 pandemic.

## Introduction

The COVID-19 pandemic has brought unprecedented disruptions to trials and drug development.^1^ The US Food and Drug Administration (FDA) has had to spend its resources reviewing SARS-CoV-2 therapies and vaccines.^2^ Despite these challenges, the FDA commissioner has stated that the FDA is “full speed ahead” in 2020 on the approval of novel cancer drugs.^3^ To assess this claim, we sought to survey all new molecular entities (NMEs) approved for cancer treatment in 2020.

## Methods

In this cross-sectional study, we reviewed the FDA Hematology/Oncology Approvals website^4^ to ascertain all hematology/oncology drugs approved in 2020. The authors (C. S. and V. P.) determined which drugs were novel, defined as having no prior FDA approval for a similar or different indication. New formulations of previously approved drugs (eg, oral formulations of previously approved intravenous formulations) were not considered to be novel drugs. We recorded the response rate, complete response rate, duration of response, progression-free survival, and overall survival as reported in the FDA prescribing information for each drug. In the event that a given approval was based on 2 different trials or 2 separate arms of a trial, we recorded the mean response between the 2 trials. The type of FDA approval (accelerated or regular) and the design of the trial were noted. Accelerated approvals require further proof of efficacy in improving overall or progression-free survival.

This study was not submitted for institutional review board approval because it did not use personal health care information and all study data are publicly available (Common Rule, 82 FR §7149).^4^ This report follows the Strengthening the Reporting of Observational Studies in Epidemiology (STROBE) reporting guideline for cross-sectional studies.

## Results

There were 18 NMEs approved for cancer treatment in 2020 as determined by the authors (Table). This was more than the 13 NMEs approved for cancer in 2019, and similar to 2018.^4^

**Table.** Effectiveness of Novel Cancer Drugs Approved by the FDA in 2020

Drug	Disease	Type of approval	Basis for approval	Response rate, %^a^	Duration of response^b^
Randomized placebo-controlled trial					
Isatuximab-irfc	Multiple myeloma	Regular	Progression-free survival in 307 patients	60	11.5 mo median progression-free survival
Margetuximab-cmkb	*ERBB2-*positive metastatic breast cancer	Regular	Progression-free survival in 536 patients	22	6.1 mo median
Ripretinib	GIST	Regular	Progression-free and overall survival in 121 patients	9	6.3 mo progression-free survival
Tucatinib	*ERBB2-*positive metastatic breast cancer	Regular	Progression-free and overall survival in 612 patients	41	7.8 mo progression-free survival
Uncontrolled, single arm phase I/II trials					
Avapritinib	GIST with *PDGFRA* exon 18 mutation	Regular	Tumor shrinkage in 43 patients	84	61% response rate lasting ≥6 mo
Belantamab mafodotin-blmf	Multiple myeloma	Accelerated	Overall response in 97 patients	31	78% at 4 mo
Brexucabtagene autoleucel	Mantle cell lymphoma	Accelerated	Tumor shrinkage in 74 patients	87	60% at 12 mo
Capmatinib	NSCLC with met exon 14 mutation	Accelerated	Tumor shrinkage in 97 patients	49	9.7-12.6 mo median
Decitabine + cedazuridine	Myelodysplastic syndrome	Regular	Response rate in 213 patients (2 trials)	60	7.5-8.7 mo median (complete responses)
Lurbinectedin	Small cell lung cancer	Accelerated	Tumor shrinkage in 105 patients	35	5.3 mo median
Naxitamab	Neuroblastoma	Accelerated	Overall response in 60 patients (2 trials)	38	23%-30% response rate lasting ≥6 mo
Pemigatinib	*FGFR2* mutant cholangiocarcinoma	Accelerated	Tumor shrinkage in 107 patients	36	9.1 mo median
Pralsetinib	*RET* fusion NSCLC + *RET* altered medullary thyroid cancer	Accelerated	Tumor shrinkage in 116 patients (lung) + 93 patients (thyroid)	65	80% at 6 mo for lung, similar for thyroid (prior platinum therapy)
Sacituzumab govitecan-hziy	Metastatic triple-negative breast cancer	Accelerated	Tumor shrinkage in 108 patients	33	7.7 mo median
Selpercatinib	*RET* fusion lung and thyroid cancers	Accelerated	Tumor shrinkage in 314 patients (3 trials)	72	76%-87% response rate lasting >6 mo
Selumetinib	plexiform neurofibromas	Regular	Tumor shrinkage in 50 patients	44	84% at 3 y
Tafasitamab-cxix	Diffuse large B cell lymphoma	Accelerated	Tumor shrinkage in 80 patients	60	21.7 mo median
Tazemetostat	*EZH2* mutant follicular lymphoma, epithelioid sarcoma	Accelerated	Response rate in 42 patients (follicular lymphoma)	69 (follicular lymphoma)	10.9 mo median (follicular lymphoma)
			Response rate in 62 patients (epithelioid sarcoma)	15 (epithelioid sarcoma)	

Abbreviations: *EZH2*, enhancer of zeste homologue 2; *ERBB2*, receptor tyrosine-protein kinase erbB-2 (formerly *HER2*); *FGFR2*, fibroblast growth factor receptor 2; GIST, gastrointestinal stromal tumor; NSCLC, non–small cell lung cancer; *PDGFRA*, platelet-derived growth factor receptor A; *RET*, ret proto-oncogene.

^a^ Response rate is the sum of partial and complete responses as determined by imaging. ^b^Duration of response is the time from drug initiation to cancer progression requiring change in treatment, cessation in treatment, or death.

Two drugs (11%) were approved based on an improvement in overall survival compared with a placebo-controlled arm. These include ripretinib, which in trial had a 15.1-month overall survival in metastatic gastrointestinal stromal tumor compared with a 6.6-month survival for patients receiving placebo, and tucatinib, which when used in combination with capecitabine and trastuzumab resulted in a mean overall survival of 21.9-months in metastatic *ERBB2* (formerly *HER2*)–positive breast cancer compared with 17.4 months in the capecitabine and trastuzumab arm. The remaining 16 novel cancer drug approvals were based on response rate or progression-free survival. Of all the novel cancer therapies approved in 2020, the median response rate (ie, partial plus complete response rate) was 49.7% (range, 9%-87%); the complete response rate ranged from 0% to 62%, with a median of 3% (Figure).

**Figure.**
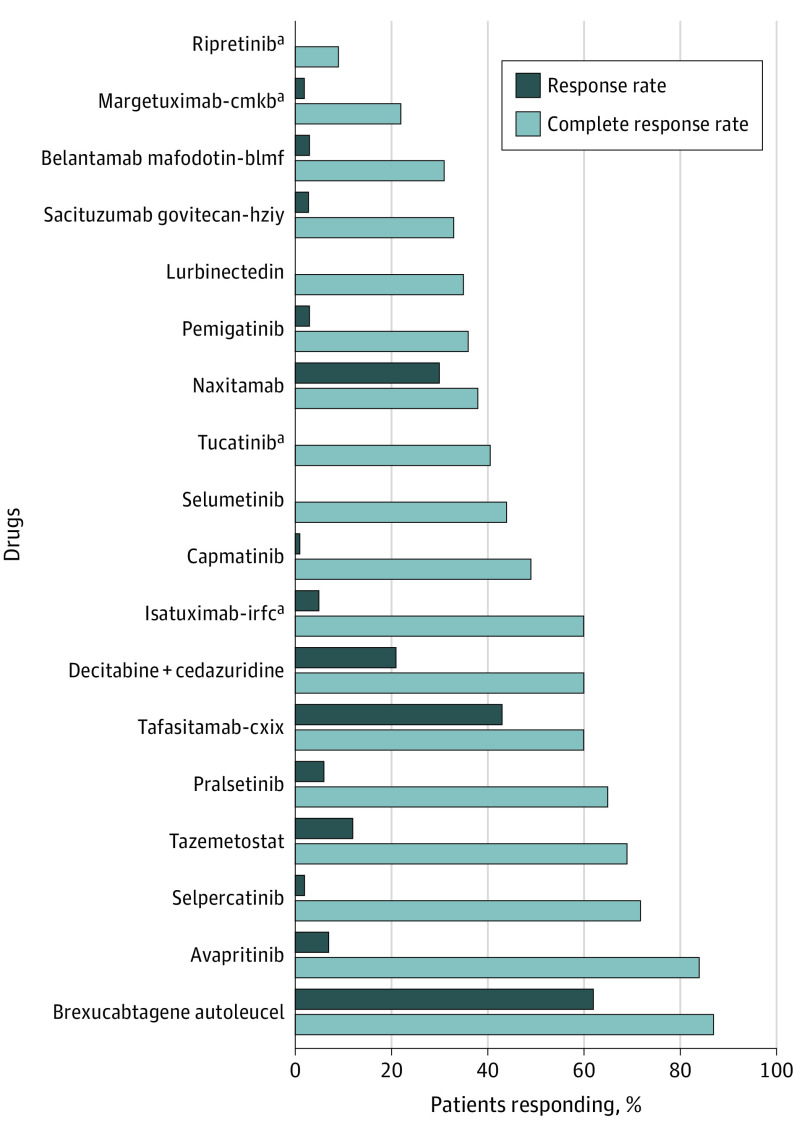
Response Rate and Complete Response Rate of Novel Cancer Drugs Approved in 2020 The overall median response rate was 49.7%. Note that for tazemetostat, only the response rate (69%) in follicular lymphoma is shown; the response rate for epithelioid sarcoma is 15%. ^a^Denotes a drug approved based upon a randomized placebo-controlled trial.

Only 4 (22%) of the approvals were based on a randomized placebo-controlled trial. The remaining 14 approvals (78%) were based on uncontrolled, single-arm phase I/II trials. Eleven of these were accelerated approvals and will require further efficacy data.

## Discussion

More NMEs were approved by the FDA for cancer in 2020 than in 2019. However, most approved NMEs were based upon surrogate end points with uncertain effects on survival and quality of life.^5^ The majority of approvals were based upon uncontrolled, single-arm clinical trials, and will require postmarket efficacy testing.^6^ Approximately half of patients given one of these novel drugs approved in 2020 will have a demonstratable tumor response. The authors acknowledge that this study is limited in that we only reviewed 1 year of FDA drug approvals. Additionally, future trial data regarding these medications may become available, rendering the observations here no longer relevant.

## References

[1] Waterhouse DM, Harvey RD, Hurley P, . Early impact of COVID-19 on the conduct of oncology clinical trials and long-term opportunities for transformation: findings from an American Society of Clinical Oncology survey. JCO Oncol Pract. 2020;16(7):417-421. doi:10.1200/OP.20.00275 32396491

[2] US Food and Drug Administration. FDA Approves First Treatment for COVID-19. Food and Drug Administration news release. Published October 22, 2020. Accessed December 4, 2020. https://www.fda.gov/news-events/press-announcements/fda-approves-first-treatment-covid-19

[3] Hahn SM (@SteveFDA). As we make progress in our understanding of #COVID19, we continue full speed ahead on our non-COVID cancer-related work, including important drug approvals. For instance, as of Oct. 30, the @US_FDA has approved 42 novel drugs including 15 new drugs to treat patients with various forms of cancer. Several of these approvals have been “firsts” for oncology. November 17, 2020. Accessed April 15, 2021. https://twitter.com/stevefda/status/1328800222449979392

[4] US Food and Drug Administration. US Food and Drug Administration hematology/oncology (cancer) approvals & safety notifications. Updated April 14, 2021. Accessed December 4, 2020. https://www.fda.gov/drugs/resources-information-approved-drugs/hematologyoncology-cancer-approvals-safety-notifications

[5] Haslam A, Hey SP, Gill J, Prasad V. A systematic review of trial-level meta-analyses measuring the strength of association between surrogate end-points and overall survival in oncology. Eur J Cancer. 2019;106:196-211. doi:10.1016/j.ejca.2018.11.012 30528804

[6] Carpenter D, Kesselheim AS, Joffe S. Reputation and precedent in the bevacizumab decision. N Engl J Med. 2011;365(2):e3. doi:10.1056/NEJMp1107201 21707383

